# Reduced primary cilia length and altered Arl13b expression are associated with deregulated chondrocyte Hedgehog signaling in alkaptonuria

**DOI:** 10.1002/jcp.25839

**Published:** 2017-03-31

**Authors:** Stephen D. Thorpe, Silvia Gambassi, Clare L. Thompson, Charmilie Chandrakumar, Annalisa Santucci, Martin M. Knight

**Affiliations:** ^1^ Institute of BioengineeringSchool of Engineering and Materials ScienceQueen Mary University of LondonLondonUnited Kingdom; ^2^ Dipartimento di BiotecnologieChimica e FarmaciaUniversità degli Studi di SienaSienaItaly

**Keywords:** actin, alkaptonuria, chondrocyte, Hedgehog signaling, primary cilium

## Abstract

Alkaptonuria (AKU) is a rare inherited disease resulting from a deficiency of the enzyme homogentisate 1,2‐dioxygenase which leads to the accumulation of homogentisic acid (HGA). AKU is characterized by severe cartilage degeneration, similar to that observed in osteoarthritis. Previous studies suggest that AKU is associated with alterations in cytoskeletal organization which could modulate primary cilia structure/function. This study investigated whether AKU is associated with changes in chondrocyte primary cilia and associated Hedgehog signaling which mediates cartilage degradation in osteoarthritis. Human articular chondrocytes were obtained from healthy and AKU donors. Additionally, healthy chondrocytes were treated with HGA to replicate AKU pathology (+HGA). Diseased cells exhibited shorter cilia with length reductions of 36% and 16% in AKU and +HGA chondrocytes respectively, when compared to healthy controls. Both AKU and +HGA chondrocytes demonstrated disruption of the usual cilia length regulation by actin contractility. Furthermore, the proportion of cilia with axoneme breaks and bulbous tips was increased in AKU chondrocytes consistent with defective regulation of ciliary trafficking. Distribution of the Hedgehog‐related protein Arl13b along the ciliary axoneme was altered such that its localization was increased at the distal tip in AKU and +HGA chondrocytes. These changes in cilia structure/trafficking in AKU and +HGA chondrocytes were associated with a complete inability to activate Hedgehog signaling in response to exogenous ligand. Thus, we suggest that altered responsiveness to Hedgehog, as a consequence of cilia dysfunction, may be a contributing factor in the development of arthropathy highlighting the cilium as a novel target in AKU.

## 1 INTRODUCTION

Alkaptonuria (AKU) is an ultra‐rare metabolic disease in which a deficiency of the enzyme homogentisate 1,2‐dioxygenase leads to the accumulation of homogentisic acid (HGA) (La Du, Zannoni, Laster, & Seegmiller, 1958); for review see Gallagher, Dillon, Sireau, Timmis, and Ranganath (2016). The oxidized HGA derivative benzoquinone acetic acid (BQA) forms a melanin‐like pigmentation known as “ochronosis,” causing dramatic tissue degeneration. The primary clinical manifestation of AKU is a severe form of early onset arthropathy, characterized by articular cartilage degeneration (Helliwell, Gallagher, & Ranganath, 2008; Mannoni et al., 2004). Within the joint, articular cartilage covers the bone surfaces where it has a protective, low friction, load distributing function (Mow, Wang, & Hung, 1999). This cartilage tissue matrix is largely comprised of a dense collagen network in which the highly hydrated glycosaminoglycan, aggrecan, is immobilized. Chondrocytes reside within this matrix and are responsible for maintaining tissue homeostasis in response to mechanical cues. Disruption of this process results in the degenerative disease osteoarthritis (OA), which is characterized by progressive failure of the articular cartilage accompanied by changes in the synovium and subchondral bone that result in loss of mobility and significant pain (Goldring & Goldring, 2007). Despite being the world's most common form of arthropathy, there is currently no treatment to prevent OA.

Many similarities between the pathogenesis of AKU and OA are apparent. A significant inflammatory contribution is present in OA (Goldring & Otero, 2011), while it has also been shown that inflammatory cytokine expression is altered in AKU chondrocytes resulting in severe inflammation (Braconi et al., 2012; Mannoni et al., 2004). Moreover, significant protein oxidation and aggregation is observed in these cells which, in addition to promoting the production of ochronotic pigments, results in the altered expression of proteins involved in cell defence, protein folding, and cell organization (Braconi et al., 2011). In both OA and AKU tissue, chondrocytes undergo a particular form of cell death termed chondroptosis (Millucci et al., 2015; Perez, Luna, Rojas, & Kouri, 2005). Chondrocytes first appear enlarged and hypertrophic with a prominent Golgi apparatus, characterized by swollen cisternae. This organelle is structurally and functionally connected with the cytoskeleton, as microtubules, and actin filaments are fundamental for the maintenance of its structural integrity (Thyberg & Moskalewski, 1999; Valderrama et al., 1998). It is well established that the cytoskeleton is disrupted in OA chondrocytes (Capin‐Gutierrez, Talamas‐Rohana, Gonzalez‐Robles, Lavalle‐Montalvo, & Kouri, 2004; Holloway et al., 2004; Haudenschild et al., 2011). Similarly, Geminiani et al. (2016) recently reported that both actin and microtubule cytoskeletal elements are disordered in AKU chondrocytes. In addition to alterations in cell shape, actin stress fibre formation is increased in AKU chondrocytes accompanied by the formation of actin bundles in the nuclear periphery (Geminiani et al., 2016). The disruption of actin organization and dynamics may have consequences for the maintenance of chondrocyte phenotype (Brown & Benya, 1988), mechanotransduction (Ohashi, Hagiwara, Bader, & Knight, 2006; Wright et al., 1997), and may also influence chondrocyte function through the primary cilium.

The primary cilium is a singular, immotile organelle present in the majority of cells during interphase. It consists of a microtubule‐based axoneme covered by a specialized plasma membrane; for review see Satir & Christensen (2008). This tubulin‐based structure is involved in many biological processes, including differentiation, and vertebrate development (Huangfu et al., 2003; Tummala, Arnsdorf, & Jacobs, 2010), cell cycle control (Tucker, Pardee, & Fujiwara, 1979), cancer signaling (Reilova‐Velez and Seiler, 1984; Wong et al., 2009), sensory function and migration (Schneider et al., 2005), and mechano‐signaling (Khayyeri, Barreto, & Lacroix, 2015; Praetorius & Spring, 2001; Wann et al., 2012). The cilium is assembled and maintained by a process called intraflagellar transport (IFT), a microtubule‐based motility present in the axoneme. IFT transports axonemal precursors and signaling proteins along the length of the cilium toward the distal tip and returns them to the basal body (Qin, Diener, Geimer, Cole, & Rosenbaum, 2004). This mechanism of transport is essential not only for maintenance of cilia structure but for the regulation of cilia‐mediated signaling pathways such as Hedgehog (Huangfu et al., 2003), Wnt (Ross et al., 2005), and TGF (Clement, Ajbro, Koefoed, Vestergaard, & Veland, 2013). Alterations in IFT lead to disruption of cilia structure and function and are responsible for a group of related disorders termed ciliopathies (Waters & Beales, 2011). The regulation of ciliogenesis is dependent on a number of actin‐regulatory molecules (Kim et al., 2010). Moreover, the regulation of primary cilia length has been linked to cell shape and the level of actin contractility such that greater actin tension is associated with cilia shortening (McMurray et al., 2013; Pitaval, Tseng, Bornens, & Thery, 2010). Cilia length modifications have been reported in several pathological conditions including OA where an increase in axoneme length is observed (McGlashan, Cluett, Jensen, & Poole, 2008).

Hedgehog signaling is regulated through the primary cilium (Corbit et al., 2005; Huangfu & Anderson, 2005; Rohatgi, Milenkovic, & Scott, 2007). Binding of Hedgehog ligands to the receptor Patched (Ptch1) triggers the IFT‐dependent accumulation of the transmembrane protein Smoothened (Smo) within the cilium (Corbit et al., 2005). This process is mediated by several proteins, among which is the GTPase Arl13b (Caspary, Larkins, & Anderson, 2007; Larkins, Aviles, East, Kahn, & Caspary, 2011). Smo ciliary distribution regulates the activity of transcription factors belonging to the Gli family (Gli1, Gli2, Gli3), the effectors of this pathway (Buttitta, Mo, Hui, & Fan, 2003). Non‐canonical Hedgehog signaling can also regulate actin cytoskeleton organization via the RhoA pathway, independent of transcription (Polizio et al., 2011; Renault et al., 2010; Sasaki, Kurisu, & Kengaku, 2010). Recent studies have demonstrated that chondrocyte Hedgehog signaling can be influenced by alterations in cilia length (Thompson, Chapple, & Knight, 2014; Thompson, Wiles, Poole, & Knight, 2016) as the size of this signaling compartment is expected to influence the rate at which proteins are delivered to, or removed from, the cilia tip (Engel, Ludington, & Marshall, 2009; Ludington, Wemmer, Lechtreck, Witman, & Marshall, 2013). Given its association with cilia length, actin cytoskeletal organization may be implicated in aberrant Hedgehog signaling. Several studies have now identified activated Hedgehog signaling as an important pathological factor in OA (Lin et al., 2009; Wei et al., 2012). Activation of this pathway in osteoarthritic cartilage promotes chondrocyte hypertrophy and matrix catabolism in vivo (Lin et al., 2009; Wei et al., 2012). However, the contribution of Hedgehog signaling to arthropathy in AKU has not previously been reported.

This study aims to investigate the effects of HGA accumulation in AKU on primary cilia structure and Hedgehog signaling. In addition to chondrocytes isolated from AKU donor cartilage, we treated healthy donor chondrocytes with HGA (+HGA) to replicate the disease state. Given the paucity of AKU disease tissue, this model of AKU with HGA addition has been implemented in vivo through direct injection into rabbit knee joints and chick embryos (Moran & Yunis, 1962), and more widely in vitro in cells from tissues including cartilage and the nervous system (Bernardini et al., 2015; Braconi, Millucci, Bernardini, & Santucci, 2015; Mistry, Jackson, Bukhari, & Taylor, 2015). These studies are the first to demonstrate that primary cilia length and prevalence are reduced in AKU chondrocytes, and chondrocytes treated with HGA, relative to healthy control cells. Consistent with previous reports we show that actin organization is altered in AKU and HGA treated chondrocytes and report that actin‐dependent cilia length regulation is disrupted. This is accompanied by altered cilia structure and trafficking such that there is an increase in the proportion of cilia exhibiting axoneme breaks and bulbous tips in AKU cells. Super resolution microscopy reveals an increase in the volume of cilioplasm at the ciliary tip which is associated with accumulation of the Hedgehog‐related protein Arl13b within the tip region. Furthermore, we identify that AKU and HGA treated cells exhibit a complete inability to activate Hedgehog signaling in response to exogenous ligands, which we propose may be the result of the observed dysfunction of cilia structure and trafficking.

## 2 MATERIALS AND METHODS

### 2.1 Isolation and culture of human articular chondrocytes

Human articular chondrocytes were obtained from knee cartilage of healthy donors and those with alkaptonuria (AKU), with full local ethics approval (CEL AOUS July 21, 2010). Chondrocytes were isolated by enzymatic digestion as previously described (Braconi et al., 2012) and cultured in Dulbecco's Modified Eagle Medium (DMEM; Life Technologies, Paisley, UK) supplemented with 10% foetal bovine serum (FBS) and penicillin (100 U/ml)—streptomycin (100 μg/ml; all Sigma–Aldrich, Dorset, UK). To induce an AKU‐like state, healthy chondrocytes were also treated with 66 μM HGA (Sigma–Aldrich) for 7 days (+HGA). Culture media was exchanged every 2–3 days and cultures were maintained at 37°C and 5% CO_2_. Chondrocytes were seeded at approx. 10 × 10^3^ cells/cm^2^ onto serum coated glass coverslips in 24‐well plates for immunofluorescence experiments, and in 6‐well plates for gene expression studies. To disrupt actin stress fibre contractility, chondrocytes were treated with the Rho‐associated protein kinase (ROCK) inhibitor Y27632 (Y27, 10 μM; Sigma–Aldrich) in the absence of FBS for 24 hr and fixed.

### 2.2 Immunofluorescent staining

Confluent chondrocytes were serum starved (0% FBS) for 24 hr prior to fixation in 4% paraformaldehyde for 10 min. Cells were permeabilized with 0.5% Triton X–100 in phosphate buffered saline (PBS) for 5 min and blocked with 5% goat serum in 0.1% bovine serum albumin‐PBS (0.1% BSA‐PBS; all Sigma–Aldrich) for 1 hr. Cells were incubated with primary antibodies in 0.1% BSA‐PBS at 4°C overnight. Mouse monoclonal anti‐acetylated α‐tubulin (clone 611B‐1, 1:2000; Sigma–Aldrich, Cat# T7451, RRID: AB_609894) and rabbit polyclonal anti‐Arl13b (1:2000; Proteintech, Manchester, UK, Cat# 17711‐1‐AP, RRID: AB_2060867) were used for the detection of the ciliary axoneme. Rabbit polyclonal anti‐pericentrin (1:500; Abcam, Cambridge, UK, Cat# ab4448, RRID: AB_304461) was used alongside anti‐acetylated α‐tubulin for the detection of centriole‐derived basal bodies. Following repeated washing in 0.1% BSA‐PBS, cells were incubated with appropriate Alexa Fluor‐conjugated secondary antibodies (1:1000; Life Technologies) for 1 hr at room temperature. F‐actin was detected using CruzFluor 488‐conjugated phalloidin (1:1000; Santa Cruz Biotechnology, Heidelberg, Germany, Cat# sc‐363791, RRID: AB_2631056) in 1% BSA‐PBS and nuclei were detected with 1 μg/ml 4′,6‐diamidino‐2‐phenylindole (DAPI; Sigma–Aldrich) in PBS. Following washes in PBS, coverslips were mounted with Fluoromount‐G (Cambridge Bioscience, Cambridge, UK).

### 2.3 Confocal and super‐resolution microscopy and image analysis

For primary cilia length measurement, samples were imaged using a Leica TCS SP2 confocal microscope with a ×63 1.4 NA objective (Leica Microsystems, Milton Keynes, UK). Confocal z‐stacks were generated containing the entire cell depth with a voxel size of 116 × 116 × 500 nm in x‐y‐z. The maximum intensity projection in the z‐axis was used for the measurement of cilia length and assessment of morphology using ImageJ software (NIH, Bethesda, MD, RRID: SCR_003070). For primary cilia prevalence, samples were imaged using a Leica DMI4000B epifluorescent microscope with a ×63 1.25 NA objective (Leica Microsystems). The proportion of ciliated cells was determined across multiple fields of view for each condition. Structural abnormalities were manually assessed on acetylated α‐tubulin stained cilia as a proportion of total cilia for each condition. Cilia tips were defined as the region distal to pericentrin staining of the basal body region (Fig. 3A). Bulbous tipped cilia were defined as having a tip diameter >1.5 × diameter at axoneme midpoint. Fragmented cilia were defined as cilia with clear breaks in acetylated α‐tubulin staining along the axoneme. Examples are presented in Figure 3A.

Arl13b and acetylated α‐tubulin stained cilia were imaged using super‐resolution structured illumination microscopy (SR‐SIM). Samples were imaged on a Zeiss 710 ELYRA PS.1 microscope with a ×63 1.4 NA objective (Carl Zeiss, Cambridge, UK). SR‐SIM processing and channel alignment were performed to obtain a voxel size of 25 × 25 × 100 nm in x‐y‐z. Maximum intensity projections in the z‐axis were used to generate intensity profiles perpendicular to the axoneme close to the base and tip of individual primary cilia as shown in Figure 3D. Cilia diameter was assessed from Arl13b and acetylated α‐tubulin signals as full width at half maximum of the intensity profile; representatives of which are shown in Figure 3E. Mean and integrated intensity of each marker was assessed for each profile at both the base and tip of the cilium.

### 2.4 Evaluation of Hedgehog pathway gene expression in response to ligand stimulation

The expression of the Hedgehog target proteins GLI1 and PTCH1 is increased upon pathway activation (Buttitta et al., 2003; Dai et al., 1999). Gene expression was evaluated in untreated chondrocytes from the three groups (Healthy, AKU, and +HGA) and chondrocytes from each group treated for 24 hr with 1 µg/ml recombinant Indian Hedgehog (Ihh; R&D Systems, Abingdon, UK) to measure pathway activity. Total RNA was extracted from isolated human chondrocytes cultured in single wells using an RNeasyKit and converted to cDNA using the QuantiTect Reverse Transcription Kit (both Qiagen, Manchester, UK) according to the manufacturer's instructions. Both RNA and cDNA were quantified using the Nanodrop ND‐1000 spectrophotometer (LabTech, East Sussex, UK). Quantitative real‐time PCR reactions were performed in 10 µl volumes containing 1 µl cDNA (diluted 1:2), 5 µl KAPA SYBR® FAST Universal 2× qPCR Master Mix containing SYBR‐green dye and ROX reference dye (KAPA Biosystems, London, UK), and 1 µl optimized primer pairs (Table 1). An annealing temperature of 60°C was used for all PCR reactions and fluorescence data was collected using the MX3000P QPCR instrument (Agilent Technologies, Cheshire, UK). Samples were run in triplicate to minimise pipetting errors. Data was analyzed using the relative standard curve method and target genes were normalized to 18s RNA.

**Table 1:** Primer sequences used in qPCR

Gene	Sequence
18S RNA	F‐CGGCTACCACATCCAAGGAA
	R‐AGCTGGAATTACCGCGGC
PTCH1	F‐GGGTGGCACAGTCAAGAACAG
	R‐TACCCCTTGAAGTGCTCGTACA
GLI1	F‐GCGTTGTAGAGAGGTAACCC
	R‐TGATGAAAGCTACGAGGGAG

### 2.5 Statistical analysis

Statistical analyses were performed using Minitab 17 software (Minitab, Coventry, UK, RRID: SCR_014483). When data sets adhered to a normal distribution, two sample *t*‐test, or a general linear model for analysis of variance with Fisher tests for multiple comparisons were used. For non‐parametric data sets, Mann–Whitney tests were used to compare conditions. Data quoted in the text are presented as mean ± s.e.m. Details of specific statistical tests and *n* values can be found in the figure legends.

## 3 RESULTS

### 3.1 F‐actin cytoskeleton is altered in AKU chondrocytes

**Figure 1:**
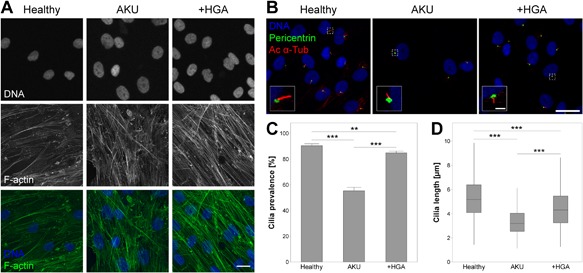
Alkaptonuria (AKU) alters articular chondrocyte F‐actin cytoskeleton, and primary cilia prevalence and length. (A) Representative F‐actin cytoskeletal organization in healthy, AKU and homogentisic acid treated (+HGA) chondrocytes, stained for F‐actin (green), and cell nuclei (blue). Scale bar 20 μm. (B) Representative confocal maximum intensity z‐projections of primary cilia from healthy AKU and +HGA chondrocytes immunofluorescently stained for acetylated α‐tubulin (Ac α‐Tub; red) and pericentrin (green). Scale bar 20 μm, inset scale bar 2 μm. (C) Primary cilia prevalence in populations of healthy, AKU and +HGA chondrocytes. Mean ± s.e.m, *n* ≥ 23 random fields of view, Mann–Whitney tests: **p < 0.01, ***p < 0.001. (D) Primary cilia length in healthy, AKU and +HGA chondrocytes; boxes represent median and interquartile range, with whiskers extending to 1.5× interquartile range or the max/min data points, *n* ≥ 316 cilia, General linear model with fisher pairwise comparisons: ***p < 0.001

Chondrocytes isolated from healthy donors exhibited some faint stress fibre formation with minimal formation of f‐actin aggregates (Fig. 1A). In contrast, AKU and HGA treated (+HGA) chondrocytes exhibited an increased intensity of f‐actin labeling with more stress fibres present and increased incidence of punctate f‐actin aggregates, suggesting a more contractile phenotype (Fig. 1A).

### 3.2 Chondrocyte primary cilia length and prevalence is reduced in AKU

Immunofluorescent labelling of primary cilia (Fig. 1B) revealed that the proportion of cells exhibiting a cilium was dramatically reduced from 90.4 ± 1.5% in healthy control cells to 55.2 ± 2.9% in AKU chondrocytes (Fig. 1C). Moreover, cilia length was significantly reduced in these cells relative to the healthy control (Fig. 1D). Cilia prevalence and length were significantly reduced in +HGA chondrocytes, but to a lesser degree than observed in AKU cells (Fig. 1C and D).

### 3.3 Actin‐dependent regulation of cilia length is disrupted in AKU chondrocytes

**Figure 2:**
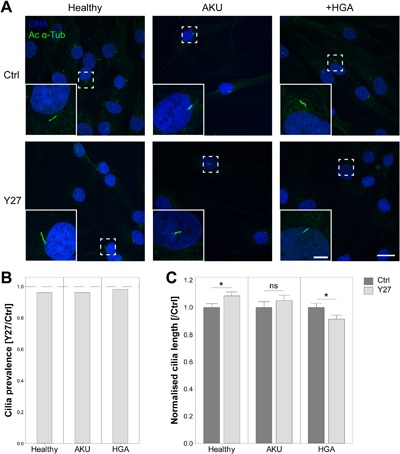
ROCK modulation of cilia length is abrogated in alkaptonuria (AKU) and homogentisic acid treated (+HGA) chondrocytes. (A) Representative confocal maximum intensity z‐projections of primary cilia from healthy, AKU and +HGA chondrocytes, with (Y27) or without (Ctrl) addition of ROCK inhibitor Y27632, immunofluorescently stained for acetylated α‐tubulin (Ac α‐Tub; green). Scale bar 20 μm, inset scale bar 5 μm. (B) Primary cilia prevalence in populations of healthy, AKU and +HGA chondrocytes treated with Y27632 (Y27) normalized to control prevalence values; *n* ≥ 120 cells. ROCK inhibition did not significantly reduce cilia prevalence. (C) Primary cilia length in healthy, AKU and +HGA chondrocytes treated with or without ROCK inhibitor (Y27) normalized to control cilia length for each cell type. Mean ± s.e.m., *n* ≥ 106 cilia, Two‐sample *T*‐test: *p < 0.05, ^ns^ *p *> 0.05

It is well established that actin contractility influences primary cilia length (Kim et al., 2010; McMurray et al., 2013; Pitaval et al., 2010). Due to the observed alterations in actin cytoskeleton (Fig. 1), we investigated the effects of ROCK inhibition on cilia length and prevalence in these cells (Fig. 2A). Chondrocytes were treated with the ROCK inhibitor Y27632 (Y27) to disrupt actin stress fibre contractility. ROCK inhibition resulted in a reduction in stress fibre formation in all cultures (data not shown). No significant effects on primary cilia prevalence were observed in control, AKU, or +HGA chondrocytes (Fig. 2B). Consistent with previous reports, primary cilia length was found to be significantly increased in healthy control chondrocytes by 8.5% from 2.55 ± 0.07 to 2.76 ± 0.07 μm (Fig. 2C). However, ROCK inhibition did not affect cilia length in AKU chondrocytes while in +HGA chondrocytes a significant reduction in length was observed (Fig. 2C). These data suggest that the regulation of cilia length through alterations in actin cytoskeletal tension and actin dynamics is disrupted in AKU and reversed in +HGA chondrocytes.

### 3.4 Primary cilia structure and protein localization is altered in AKU chondrocytes

**Figure 3:**
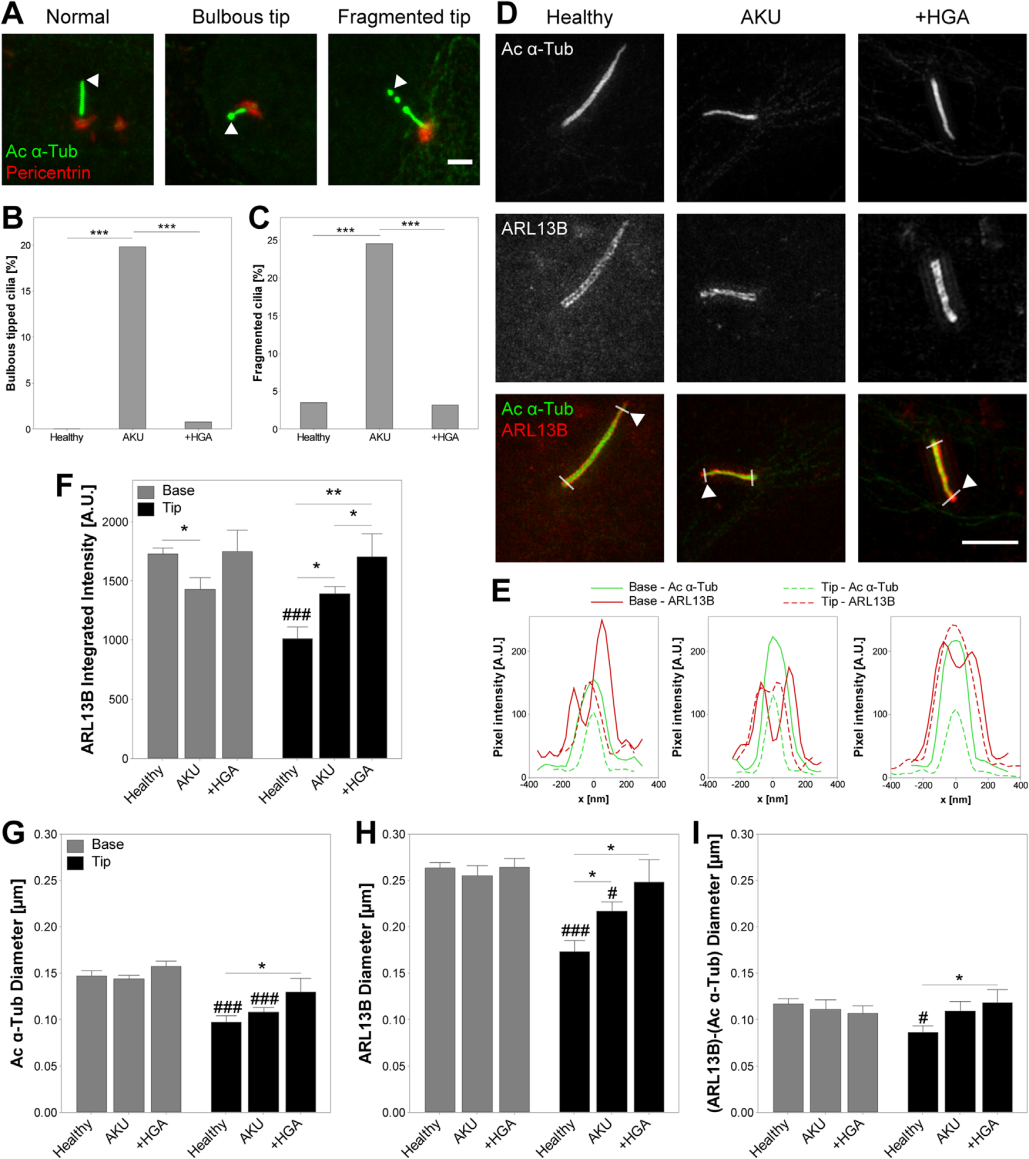
Cilia structure and Arl13b protein distribution are altered in Alkaptonuria (AKU) consistent with disrupted ciliary trafficking. (A) Representative confocal maximum intensity z‐projections of primary cilia demonstrating typical structural alterations with normal, bulbous tipped, and fragmented cilia. Chondrocytes were immunofluorescently stained for acetylated α‐tubulin (Ac α‐Tub; green) and pericentrin (red). Arrowhead: primary cilium tip. Scale bar 2 μm. (B and C) Proportions of healthy, AKU and homogentisic acid treated (+HGA) chondrocytes presenting bulbous tipped (B) or fragmented (C) primary cilia. Fisher's exact test: ***p < 0.001. (D) Representative super resolution structured illumination microscopy (SR‐SIM) maximum intensity z‐projections of representative healthy, AKU and +HGA chondrocyte primary cilia stained for Ac α‐Tub (green), and ARL13B (red). Arrowhead: primary cilium tip. Scale bar 2 μm. (E) Pixel intensity profiles at base and tip of primary cilium as indicated by white lines across cilia in D. (F) ARL13B integrated intensity at base and tip of healthy, AKU and +HGA chondrocyte primary cilia. (G–I) Diameter of ciliary components Ac α‐Tub (G), ARL13B (H), and the difference in ARL13B and Ac α‐Tub diameters (I) assessed as full width at half maximum on transverse profiles across the base and tip of primary cilia as in E. Mean ± s.e.m., *n* ≥ 11, Mann–Whitney tests: *p < 0.05, **p < 0.01*;* ^#^ *p *< 0.05, ^##^ *p *< 0.01, and ^###^ *p *< 0.001 versus Base

Primary cilia structure was found to be altered in AKU and +HGA chondrocytes compared to healthy control cells. A significant increase in the proportion of cilia exhibiting bulbous tips (Fig. 3A and B) and axoneme breaks (Fig. 3A and C) was observed in AKU suggesting that ciliary trafficking and maintenance are disrupted. +HGA chondrocytes did not exhibit an increase in morphological alterations compared to healthy controls. Consistent with these findings we report that the ciliary localization of the Hedgehog related protein Arl13b is altered, such that a significant increase in protein localization at the ciliary tip was observed in both AKU and +HGA chondrocytes, with +HGA resulting in the greatest increase (Fig. 3D–F and Supplementary Fig. S1).

Closer examination of the structure of normal (not bulbous or fragmented) primary cilia using super‐resolution structured illumination microscopy (SR‐SIM) revealed that microtubule diameter was increased at the ciliary tip in AKU and +HGA chondrocytes (Fig. 3G). Similarly, examination of ciliary diameter using the membrane protein Arl13b confirmed that total ciliary tip diameter was also increased in these cells (Fig. 3H). Consequently, we report that the amount of cilioplasm within the tip region (calculated as the difference in Arl13b and Ac‐α‐Tub diameters) is increased (Fig. 3I). This trend is consistent with the increased presence of bulbous tips in AKU chondrocytes (Fig. 3B). These data further support the hypothesis that ciliary trafficking is altered in AKU and that aspects of this ciliary phenotype can be replicated by treatment with HGA.

### 3.5 AKU chondrocytes are unresponsive to exogenous Hedgehog ligands

**Figure 4:**
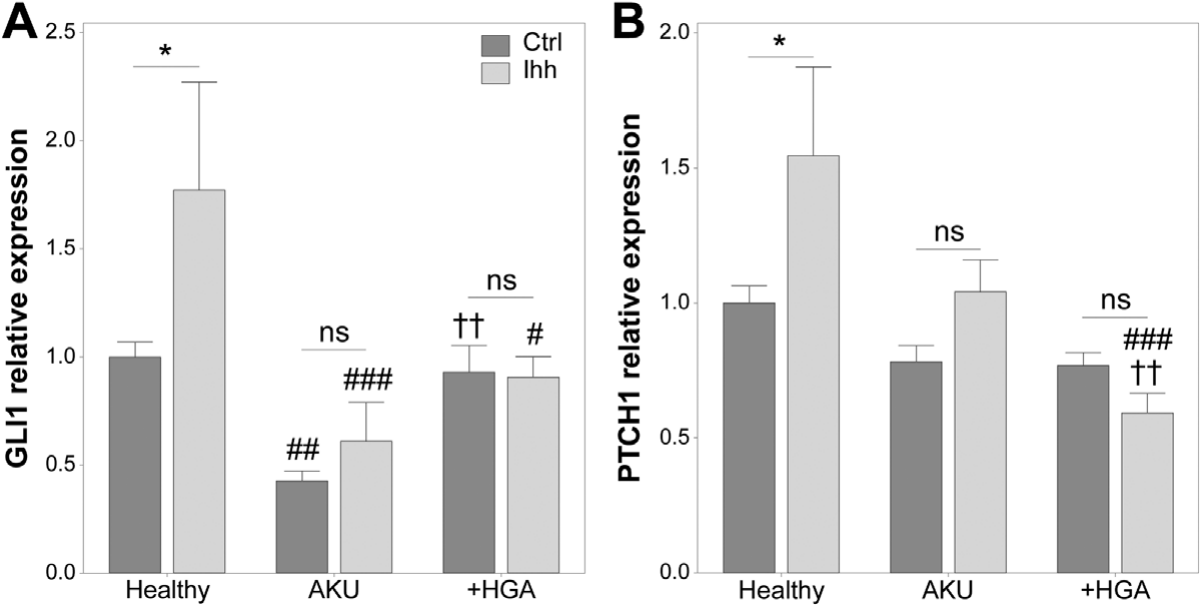
Response to indian Hedgehog ligand (Ihh) addition is abrogated in Alkaptonuria (AKU). GLI1 (A) and PTCH1 (B) gene expression in healthy, AKU and homogentisic acid treated (+HGA) chondrocytes relative to endogenous control gene GAPDH and normalized to healthy Ctrl. Mean ± s.e.m., *n* ≥ 3, general linear model with fisher pairwise comparisons: *p < 0.05, ^ns^ *p* > 0.05*;* ^#^ *p* < 0.05, ^##^ *p* < 0.01 and ^###^ *p* < 0.001 versus Healthy; ^††^ *p* < 0.01 versus AKU

Arl13b is a key regulator of the ciliary trafficking of Hedgehog proteins (Caspary et al., 2007; Larkins et al., 2011; Mariani et al., 2016). Moreover, we have recently demonstrated that alterations in cilia length and Arl13b cilia localization are associated with altered Hedgehog signaling in chondrocytes (Thompson et al., 2016). Hedgehog signaling was therefore, examined in AKU and +HGA chondrocytes under basal conditions and in response to recombinant Indian Hedgehog (Ihh) using real time PCR. Under basal conditions, the mRNA levels of GLI1 were significantly decreased in AKU but not +HGA chondrocytes relative to healthy control cells (Fig. 4A) while PTCH1 expression was not altered (Fig. 4B). In healthy control cells, expression of GLI1 and PTCH1 were significantly increased in response to Ihh treatment for 24 hr relative to untreated cells by 1.77‐ and 1.54‐fold, respectively (Fig. 4). By contrast the expression of GLI1 and PTCH1 were not significantly altered in response to Ihh in AKU or +HGA chondrocytes, demonstrating that these cells are unresponsive to ligand treatment.

## 4 DISCUSSION

These studies are the first to show that primary cilia structure and function are disrupted in the ultra‐rare metabolic disorder AKU, and that aspects of this cilia phenotype can be replicated by treatment with HGA. Subsequent studies by the authors have investigated the effect of different Hedgehog pathway antagonists on cilia structure and Hedgehog signaling in HGA treated chondrocytes. In the present study, we report variations in primary cilia prevalence and length for AKU chondrocytes which may be associated with alterations in the organization of actin (Fig. 1) and other cytoskeletal components (Geminiani et al., 2016). The filamentous microtubule structure is transformed in AKU chondrocytes, forming aggregates at the cell periphery (Geminiani et al., 2016). Furthermore, the vimentin network appears granular in these cells resulting in alterations in cell shape and cell shrinkage. Previous studies have also shown that AKU induces 4‐HNE expression and lipid peroxidation which alters microtubule function (Neely, Boutte, Milatovic, & Montine, 2005; Neely et al., 1999). Interestingly in this study, we demonstrate that the normal regulation of cilia length through the contractility of the actin cytoskeleton is disrupted in AKU cells.

It is worth noting that the cilium itself can regulate cytoskeletal organization. Hypomorphic mutation of IFT88 results in a reduction in cortical actin organization and cell stiffness in articular chondrocytes (Wang et al., 2016). Additionally, mutation of several components of the BBSome (a complex of Bardet‐Biedl syndrome (BBS) proteins involved in cilia formation) influence actin polymerization and have been shown to produce dramatic alterations in stress fibre organization that impact on cell migration, adhesion, and division in other cell types (Hernandez‐Hernandez et al., 2013). Thus, it is also possible that changes in actin cytoskeletal organization in AKU chondrocytes are downstream of alterations in primary cilia structure and trafficking.

In addition to ciliary shortening, other structural changes to the axoneme were observed. In healthy cells, primary cilia diameter, as measured from microtubule staining, typically decreases toward the ciliary tip. Electron micrographs have revealed that this is due to the conversion of microtubule doublets to singlets in the distal portion of the axoneme (Silverman & Leroux, 2009; Tanuma & Ohata, 1978; Wen, Soifer, & Wisniewski, 1982). Indeed, it is suggested that these singlet microtubules themselves have dedicated motors for transporting proteins along this distal region of the axoneme (Silverman & Leroux, 2009). In AKU and +HGA chondrocytes, ciliary diameter did not decrease to the same extent as healthy controls, perhaps suggesting more doublet microtubules extend to the tip in these shorter cilia. Furthermore, thickening of the cilium was observed in AKU cells, as measured from the ciliary membrane marker Arl13b, and an increased proportion of cilia exhibited swollen or bulbous tips (Fig. 3). Together these findings are consistent with disrupted ciliary trafficking in AKU resulting in reduced cilia length and accumulation of Arl13b at the cilia tip.

Due to the shared nature of the IFT system, alterations in ciliary trafficking in AKU would consequently influence the import/export of ciliary proteins and therefore, impact upon the dynamics of cilia signaling. This may provide an explanation for the abolition of Hedgehog signaling observed in AKU and +HGA chondrocytes. However, the relationship between cilia length and Hedgehog signaling is complex. The current literature suggests that cilia—in a state of disassembly due to reduced trafficking—will have reduced Hedgehog signaling associated with shorter cilia. However, cells with a stable short cilium that have achieved their “set length” will have an increased rate of IFT delivery and hence exhibit increased Hedgehog signaling. This relationship is demonstrated by our previous work showing that loading‐induced disassembly of primary cilia results in Hedgehog inhibition (Thompson et al., 2014), while in separate studies, cells induced to have longer cilia also have reduced Hedgehog signaling (Cruz et al., 2010; Mahjoub & Stearns, 2012; Thompson et al., 2016). Furthermore, complete cilia loss results in over activation of the pathway (Huangfu & Anderson, 2005; Huangfu et al., 2003). Thus, our data presented here suggests that reduced cilia length in AKU and +HGA chondrocytes is most likely the direct result of disrupted ciliary trafficking which also supresses ligand‐induced Hedgehog signaling.

An increase in the proportion of cilia that exhibited fragmentation within the distal region of the axoneme was observed in AKU chondrocytes (Fig. 3C). It has been postulated that this fragmentation may represent ciliary ectosomes containing molecules for long distance intercellular signaling (Hogan et al., 2009; Nager et al., 2017). Alternatively, this vesicle shedding may function as a means to remove excess or unwanted ciliary proteins as a mechanism of disposal and thus modulate cilia‐dependent signaling pathways; for review see Wood and Rosenbaum (2015). Indeed, the latter is consistent with both a reduction in Hedgehog signaling and the reduced cilia length observed in this study and therefore, may represent an alternative hypothesis for the observations reported in this study.

Upon activation of the Hedgehog signaling pathway, Gli proteins are trafficked to the ciliary tip where a complex of proteins, including the kinesin‐4 protein Kif7, is localized and regulates Gli activity (He et al., 2014). Mislocalization of these tip proteins results in pathway disruption. One possibility therefore, is that within the shorter cilia of AKU cells, the formation of this tip complex is disrupted resulting in defective Hedgehog signaling. The effect of disease‐dependent disruption of cilia on the Hedgehog related protein Arl13b was also analyzed and cilia localization found to be altered. This observation may have important consequences for downstream signaling as Arl13b is required for the ciliary traffic of Ptch1, Smo, Gli2, Gli3, and other Shh‐associated proteins in response to ligand stimulation (Larkins et al., 2011). Loss of this protein can also influence basal Hedgehog signaling by promoting the constitutive production of Gli activator proteins, so that an increased ciliary concentration of Arl13b, as observed for AKU cells, might be expected to result in altered levels of Gli1 (Caspary et al., 2007). Of note, Arl13b has also been implicated in cilia length control itself (Caspary et al., 2007; Lu et al., 2015); overexpression of Arl13b results in abnormally long cilia, and increased levels of Arl13b induce protrusion of the ciliary membrane which is rapidly followed by extension of axonemal microtubules (Lu et al., 2015). In the absence of Arl13b, cilia are short and exhibit structural defects in the axonemal microtubules (Caspary et al., 2007), suggesting a complex inter‐relationship.

Throughout this study, in addition to AKU cells from patient donors, we have also used a well‐established in vitro model recapitulating aspects of AKU through supplementation with HGA (+HGA) (Braconi et al., 2015) to investigate the effects of HGA accumulation on primary cilia. While results for +HGA chondrocytes were broadly similar to those observed for AKU chondrocytes, a reduced ciliary response was apparent in the +HGA model despite comparable inhibition of Hedgehog signaling (Figs. 1, 2, 3, 4). This difference is likely the result of the extended exposure of AKU cells to increased levels of HGA. In our model, treatment was only conducted for 7 days which is sufficient to reduce proliferation and proteoglycan production, and induce significant deposition of ochronotic pigment (Tinti et al., 2011). However, while deposition of serum amyloid A (SAA), a severe complication of chronic inflammatory conditions such as rheumatoid arthritis and AKU, is present after HGA treatment, this is to a far lesser extent than in AKU cells (Geminiani et al., 2016). This, combined with our data indicating significant differences between AKU and +HGA chondrocytes, demonstrates that while HGA treatment can replicate many facets of AKU pathology, it is an incomplete disease model.

Several studies have now shown that very minor changes in cilia structure and trafficking can have dramatic effects upon functionality (Cruz et al., 2010; Thompson et al., 2014; Thompson et al., 2016; Tran et al., 2008). Thus, while the cilia disruption in +HGA cells does not influence cilia structure to the same extent as in AKU cells, it is sufficient to considerably disrupt function. Indeed, despite a much smaller reduction in length relative to the healthy control for +HGA when compared to AKU (a cilia length reduction of 16% vs. 36%, respectively, Fig. 1D), greater accumulation of Arl13b at the ciliary tip was in fact observed in +HGA chondrocytes (Fig. 3F).

It is widely accepted that the disruption of Hedgehog signaling in articular cartilage leads to the development of OA (Li, Cai, Hu, Wu, & Li, 2015b; Lin et al., 2009; Wei et al., 2012). Moreover, the level of activation and aberrant expression of Ihh protein correlates with disease severity (Lin et al., 2009; Shuang et al., 2015). Targeting Hedgehog signaling in disease models has shown some efficacy in the prevention of articular cartilage degeneration (Li, Cai, & Ding, 2015a; Lin et al., 2009), osteophyte formation (Ruiz‐Heiland et al., 2012) and synovitis, and synovial hyperplasia (Zhu et al., 2017). Given the dependence of Hedgehog signaling upon primary cilia integrity and the disruptions to cilia structure reported in this study, the concept of “ciliotherapy” (targeting disease pathways through the cilium) is one that could be readily applied to the treatment of cartilage degeneration not only in OA, but in the arthropathy associated with AKU.

## Supporting information

Additional supporting information may be found in the online version of this article at the publisher's web‐site.

Supporting Information S1.
