# Supplementary material for: Reduced primary cilia length and altered Arl13b expression are associated with deregulated chondrocyte Hedgehog signaling in alkaptonuria
Source: J Cell Physiol. 2017 Mar 31;232(9):2407–17. doi: 10.1002/jcp.25839 (PMC5484994; doi:10.1002/jcp.25839)
Supplement: Supplementary file 1 — Supporting Information S1. [file JCP-232-2407-s001.pdf]

Supplementary Material

Reduced primary cilia length and altered Arl13b expression are associated with deregulated chondrocyte hedgehog signalling in alkaptonuria

Stephen D Thorpe[1], Silvia Gambassi[2], Clare L Thompson[1], Charmilie Chandrakumar[1], Annalisa Santucci[2]†, Martin M Knight[1]*

[1]Institute of Bioengineering, School of Engineering and Materials Science, Queen Mary University of London, London, UK

[2]Dipartimento di Biotecnologie, Chimica e Farmacia, Università degli Studi di Siena, Siena, Italy

†Correspondence to: Annalisa Santucci
Address: Università degli Studi di Siena, Dipartimento di Biotecnologie, Chimica e Farmacia, Via Aldo Moro 2, 53100 Siena, Italy
E-mail: annalisa.santucci@unisi.it
Tel: +390577-234958
Fax: +390577-234954

*Correspondence to: Martin M Knight
Address: School of Engineering and Materials Science, Queen Mary University of London, Mile End Road, London E1 4NS, United Kingdom
Email: m.m.knight@qmul.ac.uk
Tel: +44 (0)20 7882 8868

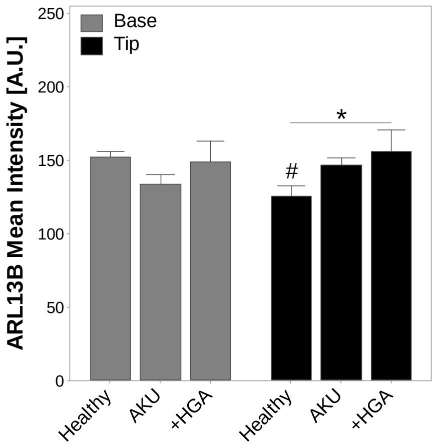

Supplementary Fig. 1. Differential mean ARL13B intensity at base and tip of primary cilium. Mean intensity of ARL13B was assessed on immunofluorescently labelled primary cilia from Healthy, Alkaptonuria (AKU) and homogentisic acid treated (+HGA) chondrocytes imaged using super resolution structural illumination microscopy; mean±s.e.m., $n \geq 11$ primary cilia, General Linear Model with Fisher pairwise comparisons: $^{*}P<0.05$, $^{\#}P<0.05$ vs. Base.
